# Prognosis of chronic low back pain: design of an inception cohort study

**DOI:** 10.1186/1471-2474-8-11

**Published:** 2007-02-08

**Authors:** Luciola da Cunha Menezes Costa, Nicholas Henschke, Christopher G Maher, Kathryn M Refshauge, Robert D Herbert, James H McAuley, Anurina Das, Leonardo OP Costa

**Affiliations:** 1 Back Pain Research Group, Discipline of Physiotherapy, University of Sydney, PO Box 170, Lidcombe NSW 1825, Australia; 2 Discipline of Physiotherapy, University of Sydney, PO Box 170, Lidcombe NSW 1825, Australia

## Abstract

**Background:**

Although clinical guidelines generally portray chronic low back pain as a condition with a poor prognosis this portrayal is based on studies of potentially unrepresentative survival cohorts. The aim of this study is to describe the prognosis of an inception cohort of people with chronic low back pain presenting for primary care.

**Methods/Design:**

The study will be an inception cohort study with one year follow-up. Participants are drawn from a cohort of consecutive patients presenting with acute low back pain (less than 2 weeks duration) to primary care clinics in Sydney, Australia. Those patients who continue to experience pain at three months, and are therefore classified as having chronic back pain, are invited to participate in the current study. The cohort will be followed up by telephone at baseline, 9 months and 12 months after being diagnosed with chronic low back pain. Recovery from low back pain will be measured by sampling three different outcomes: pain intensity, interference with function due to pain, and work status. Life tables will be generated to determine the one year prognosis of chronic low back pain. Prognostic factors will be assessed using Cox regression.

**Discussion:**

This study will determine the prognosis of chronic non-specific low back pain in a representative cohort of patients sourced from primary care. The results of this study will improve understanding of chronic low back pain, allowing clinicians to provide more accurate prognostic information to their patients.

## Background

Chronic low back pain (pain of at least three months duration [1] [references]) is a significant health problem and a major cause of work absence and disability in industrialised countries. In Australia about 1 in 10 people have chronic low back pain at any one time[2].

Clinical guidelines and textbooks typically portray chronic low back pain as a condition with a poor prognosis: they indicate that recovery is unlikely and that most people are troubled by frequent recurrences or exacerbations over time [1]. However this conclusion is based on studies with suboptimal designs[3,4].

The major difficulty in designing valid studies of prognosis of chronic low back pain is in assembling a representative cohort[5]. Most studies of chronic low back pain follow survival cohorts which sample prevalent cases. Such cohorts are likely to contain an over-representation of people with longstanding disease, and may therefore provide unduly pessimistic estimates of prognosis. A stronger design involves sampling in a representative way from a population that is at risk of developing chronic low back pain and then identifying an inception cohort from incident cases.

The present study examines a sub cohort from a larger inception cohort of people with acute low back pain [6]. The cohort of people with *acute *low back pain is at risk of developing *chronic *low back pain. By examining incident cases of chronic low back pain from the larger cohort of acute low back pain we will assemble, for the first time, a representative cohort of people with chronic low back pain. The primary aim is to determine the typical course of chronic non-specific low back pain in the first year following its onset in terms of pain, disability and return to work. A secondary aim is to develop a prognostic model for chronic low back.

## Methods

This study will be nested within an existing prospective inception cohort study of the prognosis of acute low back pain[6]. Consecutive patients from the acute prognosis study who continue to report pain at 3 months will be defined as cases of chronic low back pain and will be invited to join the chronic prognosis study. The cohort will be followed up by telephone 9 months and 12 months after being diagnosed with chronic low back pain (12 and 15 months after first consulting a primary care practitioner for an episode of acute low back pain). The study design, procedures and informed consent were approved by The University of Sydney Human Ethics Committee.

### Study sample

Participants in the acute prognosis study[6] will be recruited from primary care clinics in Sydney, Australia. Clinics were chosen to achieve a range of socio-economic levels amongst participants. All practictioners (general practitioners, physiotherapists and chiropractors) will be trained in either small groups or individual sessions, they will be asked to identify all eligible patients presented at their clinics. More comprehensive information on the sampling procedure has been published[6].

Preliminary data from the acute prognosis study indicates that approximately 40% of participants are unrecovered at 3 months[6]. With ~1,000 subjects with acute low back pain to be enrolled in the acute study we anticipate enrolling ~400 subjects in the chronic prognosis study.

Subjects will be eligible to participate in the larger cohort of acute low back pain if they meet the inclusion criteria below:

• Aged 14 years or over

• Low back pain anywhere in a region bounded superiorly by T12 and inferiorly by the buttock crease with or without leg pain

• Current episode preceded by a period of at least one month without low back pain

• Patient consents to participate in study

• No serious spinal pathology (eg. cancer, spinal infection, spinal fracture, inflammatory disorder).

All participants in the acute pain cohort who still have pain 12 weeks after the onset of their symptoms will be invited to participate in the study of the cohort of patients with chronic low back pain.

### Measurement of prognostic outcomes

There is no universally accepted definition of recovery from low back pain. We will use three definitions of recovery that differ in that they depend on pain intensity, interference with function due to pain, or work status. Outcomes will be assessed at baseline, 9 months and 1 year with simple questions suitable for telephone interview and mail survey of a large cohort of patients.

The first two questions are adaptations of items 7 and 8 of the SF36 [7] (We have changed the original wording from 'bodily pain' to 'low back pain' to reflect our specific interest in low back pain). Participants will be asked:

1. "How much low back pain have you had in the past week?" (For which response options are "none", "very mild", "mild", "moderate", "severe" and "very severe".)

2. "During the past week, how much did low back pain interfere with your normal work (including both work outside the home and housework)?" (for which response options are "not at all", "a little bit", "moderately", "quite a bit" and "extremely".)

The subset of subjects who were employed at the time of onset of symptoms will be asked to rate their work status on a scale adapted from Kenny[8].

1. Full time, Full duties

2. Full time, Selected duties

3. Part time, Full duties

4. Part time, Selected duties

5. Employed, Sick leave

6. Unemployed

7. Retired or Not currently seeking paid employment

8. Maternity leave, Long service leave

9. Other, please specify

Prognostic factors are measured at the onset of the acute episode and also at the onset of chronicity. Baseline data will be collected by the treating clinician and follow-up data will be collected by the research assistant by phone or mail. The predictors are measures of socio-demographic characteristics, general health, low back pain history and psychological characteristics. To minimise burden on the participants most constructs are assessed with brief questions designed for phone administration.

More extensive measures of three additional constructs (disability[9], self-efficacy [10] and kinesiophobia [11]) will be obtained from a subset of patients. These three constructs are the dominant constructs in theories used to explain failure to recover from chronic low back pain [12,13]. All features associated to the participants measured, the timing of the measure and the measurement tool/question used are listed in Table 1 [see Additional file 1].

### Monitoring of data quality

Several mechanisms will be used to ensure study data are of high quality. The research assistant who collects the data sheets will provide feedback to practitioners if there is evidence that the protocol is not being followed. Data will be entered and double checked by two people, and inconsistencies resolved by contacting the participant where appropriate, or via consensus.

### Data analysis

Data will be collected on date of return to pre-injury work status and/or had no disability and/or had no pain, to enable construction of Survival curves. Survival curves will be used to describe the prognosis of patients with chronic non-specific low back pain presenting to primary care practitioners. Median survival times (days to recovery) will be determined for each of the three recovery measures.

Cox regression will be used to evaluate putative prognostic factors. The independent variables for the regression will be chosen from among those collected at baseline. A correlation matrix will be inspected to determine relationships between candidate variables and outcome. Variables with strong correlations (p < 0.10) will be identified and entered into the regression model. Linear regression will be used to predict continuous outcomes such as days off work. If necessary, dependent variables will be transformed so that they satisfy assumptions of normality of residuals and heteroscedasticity. If there is evidence of non-linear effects, quadratic or higher order terms will be added to the regression model.

## Discussion

Patients with chronic low back pain (i.e. low back pain of 12 weeks duration) will be recruited from the primary care professions who most frequently manage low back pain in Australia. By sampling incident cases of chronic low back pain from a larger cohort of people with acute low back pain we will be able to study a representative cohort of patients. The choice of outcome measures reflects a standardised definition for an episode of low back pain[14] proposed to lead to more uniform reporting of the course of low back pain. By measuring three dimensions of recovery (pain, disability, and work status), a complete description of the impact of low back pain can be determined. The results of this study will improve the understanding about chronic low back pain helping clinicians to guide their clinical decision making as well as to predict chronic low back pain more accurately.

The study has been designed to minimise bias. Most importantly, we will sample from an inception cohort of chronic low back pain patients, and strategies will be used to minimise loss to follow-up[5]. It is not possible to blind measurement of prognostic outcomes to the measures of prognostic factors because both rely upon participant self-report.

A particular strength of this study is that the prognostic factors related to the initial acute episode are measured at the time of the initial acute episode rather than relying upon recall. Beyond the obvious problem of poor memory introducing error or missing values there is evidence that participant's current health state biases their recall of their previous health state. With regard to recall of pain/disability intensity it has been found that errors in recall increase with time and recalled intensity is biased by current levels of pain/disability[15,16].

## Competing interests

The author(s) declare that they have no competing interests.

## Authors' contributions

All authors participated in the design of the study. LCMC, CGM and LOPC drafted the manuscript with input from the other authors. All authors read, revised and approved the final manuscript.

## Pre-publication history

The pre-publication history for this paper can be accessed here:



## Supplementary Material

Additional File 1 Table 1. Constructs measured as predictors. All features associated to the participants measured, the timing of the measure and the measurement tool/question used are listed in this file. Click here for file
