# Supplementary material for: Prognosis of chronic low back pain: design of an inception cohort study
Source: BMC Musculoskelet Disord. 2007 Feb 8;8:11. doi: 10.1186/1471-2474-8-11 (PMC1800846; doi:10.1186/1471-2474-8-11)
Supplement: Additional File 1 — Table 1. Constructs measured as predictors. All features associated to the participants measured, the timing of the measure and the measurement tool/question used are listed in this file. [file 1471-2474-8-11-S1.doc]

**Table 1. Constructs measured as predictors**

| **Features associated with participants** | **Timing of the measurement** | **Question or Measurement tool** | **Response options** | **Source** |
| --- | --- | --- | --- | --- |
| Date of Birth | Acute baseline | What year were you born? | Open-ended | [14] |
| Gender | Acute baseline | Are you? | Male  Female | [14] |
| Indigenous Australian | Acute baseline | Are you an Aboriginal or Torres Strait Islander? | Yes  No | [14] |
| Australian born | Acute baseline | Were you born in Australia? | Yes  No | [14] |
| Country of Birth | Various | What is your country of birth? | Open-ended | [14] |
| Religion | Various | Which religion do you most closely identify with? (For example: Christian, Muslim, Hindu) | Open-ended | [14] |
| Ethnicity | Various | Which ethnic group do you identify with? (For example: English, Maori, Australian, Chinese, Lebanese) | Open-ended | [14] |
| Education level | Acute baseline | What is the level of the highest qualification you have completed? | School Certificate  Higher School Certificate  Trade Certificate  Diploma  Advanced Diploma  Bachelor Degree  Postgraduate Degree  Other | [14] |
| Work status | Acute, chronic baseline, and 12 months chronic | What was your work status when pain started? | Full time, Full duties  Full time Selected duties  Part time, Full duties  Part time, Selected duties  Employed, Sick leave  Unemployed  Retired or Not currently seeking paid employment  Maternity leave, Long service leave  Other, please specify | [8] |
| Smoking | Acute and chronic baseline | Do you currently smoke? | Yes  No |  |
| Undertaking exercise | Acute and chronic baseline | At the commencement of this back pain episode were you exercising for at least 30 minutes 3 times per week or more? (exercise includes walking briskly, cycling, digging, scrubbing floor on hands and knees etc) | Yes  No | [7] |
| Perceived general health | Acute and chronic baseline | In general would you say that your health is: | Excellent  Very good  Good  Fair  Poor | [7] |
| **Features associated with acute episode** |  |  |  |  |
| Pain location | Acute baseline | Where do you have pain? | (tick all that apply)  Neck  Shoulders  Upper back  Lower back  Leg | [15] |
| Pain Intensity | Acute, chronic baseline, 9 and 12 months chronic | How much low back pain have you had during the past week? | None  Very mild  Mild  Moderate  Severe  Very severe |  |
| Previous History | Acute baseline | Have you had a previous episode of low back pain? | Yes  No |  |
|  | Acute baseline | Have you previously taken sick leave due to low back pain? | Yes  No |  |
|  | Acute baseline | Have you previously had surgery for low back pain? | Yes  No |  |
|  | Acute baseline | Was the onset of low back pain sudden? | Yes  No |  |
| Compensation status | Acute baseline | Is your back pain compensable? E.g. workers compensation, 3^rd^ party | Yes  No |  |
| Medication use | Acute and chronic baseline | Are you currently taking medication for your low back pain? | Yes  No |  |
|  | Acute and chronic baseline | What type of medication are you using? | Open ended |  |
| Significant life event |  | In the past 12 months have you had any major illness, trauma, or undergone a major surgery? | Yes  No  If yes, what? |  |
| ADL limitation | Acute baseline | In relation to the current episode, on how many days did back or leg pain force you to cut down on the things you usually do, for more than half a day? | ___ days | [16] |
|  | Acute baseline | How long ago did the present episode of low back pain begin? | Less than 2 weeks ago  2 to 3 weeks ago  3 to 4 weeks ago  4 to 5 weeks ago  5 to 6 weeks ago |  |
| Work limitation | Acute baseline | In relation to the current episode, on how many days did back or leg pain keep you from going to work or school? | ___ days | [16] |
|  | Acute and chronic baseline and 9 months chronic | How much low back pain have you had during the past 7 days? | None  Very mild  Mild  Moderate  Severe  Very severe | [7] |
|  | Acute and chronic baseline and 9 months chronic | During the past 7 days, how much did low back pain interfere with your normal work (including both work outside the home and housework)? | Not at all  A little bit  Moderately  Quite a bit  Extremely | [7, 16] |
| Psychological | Acute and chronic baseline | If you had to spend the rest of your life with the symptoms you have right now, how would you feel about it? | Very dissatisfied  Somewhat dissatisfied  Neither satisfied nor dissatisfied  Somewhat satisfied  Very satisfied | [16] |
|  | Acute and chronic baseline | Based on all the things you do to cope, or deal with your pain, on an average day, how much are you able to decrease it? | Scale 0-10  0 - Can’t decrease it at all  10 - Can decrease it completely | [15] |
|  | Acute and chronic baseline | How tense or anxious have you felt in the past 7 days? | Scale 0-10  0 - Absolutely calm and relaxed  10 - As tense and anxious as I’ve ever felt | [15] |
| Depression | Acute and chronic baseline | How much have you been bothered by feeling depressed in the past 7 days? | Scale 0-10  0 - Not at all  10 - Extremely | [15] |
|  | Acute and chronic baseline | In your view, how large is the risk that your current pain may become persistent? | Scale 0-10  0 - No risk  10 - Very large risk | [15] |
| Return to work | 9 and 12 months chronic | (i) During the past 9/12 months did you return to pre-injury work hours and duties and also stay there for a whole month?  (ii) Can you tell me the date you returned to your pre-injury work levels? | (i) Yes/no  (ii) open-ended |  |
| Return to normal ADL | 9 and 12 months chronic | (i) During the past 9/12 months did your reach the point where the back pain caused no interference at all with work AND this interference-free period last for a whole month?  (ii) Can you tell me the date when you had no interference to your work? | (i) Yes/no  (ii) open-ended |  |
| Self-Efficacy at commencement of episode of CLBP | Chronic baseline | Pain Self Efficacy Scale | 10 item scale 0-6 | [10] |
| Kinesiophobia at commencement of episode of CLBP | Chronic baseline | Tampa Scale of Kinesiophobia | 17-item scale, score range from 17-68 | [11] |
| **Features measured longitudinally** |  |  |  |  |
| Improvement in self-efficacy | 12 months chronic | Pain Self Efficacy Scale | 10-item scale, score range from 0 to 60 | [10] |
| Improvement in kinesiophobia | 12 months chronic | Tampa Scale of Kinesiophobia | 17-item scale, score range from 17-68 | [11] |
